# Supplementary material for: Pairing medical students on the wards: A multi-site analysis of pairing effect on clerkship performance
Source: PLoS One. 2022 Nov 29;17(11):e0277898. doi: 10.1371/journal.pone.0277898 (PMC9707778; doi:10.1371/journal.pone.0277898)
Supplement: Attachment — Submitted filename: Pairing medical students on the wards.docx [file pone.0277898.s001.docx]

Pairing medical students on the wards: A multi-site analysis of pairing effect on clerkship performance

**Abstract (Methods)**

………………………….to assess whether students paired together on a medical team during their Internal Medicine sub-internship affected each other’s grade.

Comment: *This is again repetition of objective*)

Comment: *What statistical tests was applied (Missing). How they were examined (might be OSCE / MCQs) is not clear.*

**Introduction** line 45

Clinical clerkships represent some of the most educational and transformative experiences in undergraduate medical education.^1^ Comment: *No reference needed*

**Statistics** Line 134 -135

To investigate whether the effect of pairing students was dependent on prior performance, we compared the observed and expected distribution of sub internship grade pairs stratified by students’ performance on their internal medicine clerkship

Comment: *It was retrospective study, they had factual data of 3^rd^ and 4^th^ year. They didn’t need; expected values of grades.*

Comments: *They need to apply assumptions whether data was normally distributed or not. This part is completely missing.* Shapiro-Wilk test, Histogram, Box Plot etc.

**Setting Line 85-87**

….which is comprised of the following 10 items:

Comments: *There were 10 variables authors could observe the effect of pairing on these variables* by comparing 3^rd^ and final year students. *If they do, it would be much better*

**Analysis** Line 96-97

Comment: *Authors had data from 3 hospital sites of Harvard Medical school. They could compare the three sites using ANOVA.*

Comment: *It is not clear whether students from 3^rd^ year and 4^th^ year were the same or different. Authors should clarify. Reading further it looks the students (3^rd^ and 4^th^ yr.) were the same. In this case Paired t test could be used to observe the difference in their grades.*

Comment: ** Chi-square test with 3 degrees of freedom *authors never write chi-square value? this is very important to mention its value. They should add two columns more in each table one for residual value and other for chi-square value*

Line 84-88

The sub-internship grade is a global rating based on the students’ clinical performance which is comprised of the following 10 items: history taking, physical examination, fund of knowledge, patient management, clinical evaluation and management skills, interpersonal skills, presentation skills, professionalism, cultural/social/systems awareness and initiative and desire to learn.

**Conclusions** - Line 261

~~Our exploratory~~ study demonstrated no significant effect of student pairing on grades in a medicine sub-internship. Comments ( it was retrospective study)

The calculated value of Chi-Square goodness of fit test is compared with the table value. If the calculated value of Chi-Square goodness of fit test is greater than the table value, we will reject the null hypothesis and conclude that there is a significant difference between the observed and the expected frequency.

More likely

P>0.05 we fail to reject the null hypothesis and we conclude that there is significant affect of pairing ….on the grading of students.

Regards

Dr Ashfaq Akram
