# Supplementary material for: Pairing medical students on the wards: A multi-site analysis of pairing effect on clerkship performance
Source: PLoS One. 2022 Nov 29;17(11):e0277898. doi: 10.1371/journal.pone.0277898 (PMC9707778; doi:10.1371/journal.pone.0277898)
Supplement: Attachment — Submitted filename: Responses.docx [file pone.0277898.s004.docx]

Thank you for the opportunity to address the reviewer’s comments which we feel have improved the manuscript.

Abstract:
1- Please explain more about the procedure of your work.

Author response: Thank you for this suggestion, we expanded the Methods section of the abstract as follows. “We examined 186 4th year student-pairs during the required medicine sub-internship at 3 hospital sites of Harvard Medical School from 2013-2017. Chi-square tests were used to determine whether pairing impacted the final clerkship grade. Subsequently we examined whether pairing impacted the sub-internship performance stratified by students’ 3rd year core medicine clerkship grade to account for prior performance.”

Reviewer comment: 2- Please write the keywords based on MeSH.

Author response: We have added the keywords as suggested

Reviewer comment: Introduction:
This section was written very well.
Methods:
3- The second sentence was not written clearly; please modify it.

Author response: We agree and have reworded this sentence as follows, “We did not examine other clerkships because students either worked alone or were paired with multiple different partners during the rotation. In addition, other required clerkships utilized shelf exams in their grading, further hindering the evaluation of the pairing effect on performance.”

Reviewer comment: 4- It would be better to write the number of paired students in the setting section.

Author response: Thank you for this suggestion. We merged the setting and participants section with the participants section first to have the number of students examined early in the methods section.

Reviewer comment: 5- Please explain more about the sub-internship grade in the setting section.

Author response: We elaborated on the sub-internship grade as follows. “Sub-internship grades were assigned by the clerkship director at each site based on written evaluations by faculty and residents who worked directly with the student. Each evaluator submitted an assessment of the student on each of the students’ clinical performance which is comprised of the following 10 items: history taking, physical examination, fund of knowledge, patient management, clinical evaluation and management skills, interpersonal skills, presentation skills, professionalism, cultural/social/systems awareness and initiative and desire to learn. The clerkship director then assigned one of the following grades based on their assessment of the written evaluations: Honors with Distinction (HD), Honors (H), Pass (P), or Fail (F). There was no written examination.

Reviewer comment: 6- It would be better to write the participants section at the beginning of the methods section.

Author response: We agree and have moved up the participants section as previously described.

Reviewer comment: Results:
7- The description of table 2 was not precise and clear.

Author response: We thank the reviewer for this observation and edited the description to state “Paired student grades for the medicine sub-internship, stratified by third year internal medicine clerkship performance.”

Reviewer comment: Discussion and Conclusion:
These sections were written well.

Author response: Thank you for these kind words
